# Comparison of Indoor Mosquito Collection Methods in the Assessment of Lymphatic Filariasis Transmission Dynamics in Mosquito Vectors in Tana River County, Kenya

**DOI:** 10.24248/EAHRJ-D-16-00401

**Published:** 2018-04-01

**Authors:** Nancy Mutanu Kinyatta, Zipporah Wangui Ng'ang'a, Luna Kamau, Jim Mwaniki Kagai

**Affiliations:** a Centre for Biotechnology Research and Development, Kenya Medical Research Institute, Nairobi, Kenya; b The South Eastern Kenya University, Kitui, Kenya

## Abstract

**Background:**

Lymphatic filariasis (LF) is a parasitic infectious disease that is transmitted by several species of mosquitoes. Diagnosis of LF is done in both human hosts and vectors. Effective mosquito collection method(s) is/are required in order to collect large numbers of mosquitoes with high chances of infectivity.

**Methods:**

In this study, 3 mosquito sampling methods were compared. Mosquitoes were collected from 6 randomly selected villages of Tana River County, Kenya. The effectiveness of CDC light traps, gravid traps, and pyrethrum spray methods in collecting mosquitoes were compared. Mosquitoes were morphologically identified into genera and species level, and mosquito dissection was done in search of microfilariae larvae to assess the infection and infectivity rates. Data was analysed by SPSS version 15.0 and analysis of variance (ANOVA).

**Results:**

A total of 1632 female mosquitoes were collected belonging to 5 mosquito genera: *Culex, Anopheles, Aedes, Mansonia*, and *Ficalbia*. The most abundant mosquito genera was *Culex*. Light traps obtained the most blood-fed mosquitoes.

**Conclusion:**

Light traps were found to be the most effective method of mosquito collection in terms of high catches and high infectivities.

## INTRODUCTION

Lymphatic filariasis (LF) is a chronic parasitic disease of public health and socioeconomic significance in tropical and subtropical countries. More than 128 million people are estimated to be infected in 83 countries worldwide, with nearly 1.2 billion people at risk. The Global Programme to Eliminate Lymphatic Filariasis was launched in 2000^1^ with the aim of interrupting LF transmission through chemotherapy and vector control. Mosquitoes are the main vectors of lymphatic filariasis parasites: the *Culex, Anopheles*, and *Aedes* species transmit *Wuchereria bancrofti* and the *Mansonia* and *Anopheles* species are involved in the transmission of *Brugia malayi*.^2^

The development and transmission of the LF parasite follows this cycle. Upon feeding from infected blood, mosquitoes acquire microfilariae (L1) from host circulation system; development of the parasite takes place in the mosquito (L2) to the infective stage (L3). The monitoring of a control intervention strategy involves assessment of mosquitoes carrying microfilariae developing larvae stages (L1 to L3) or having the human infective stage (larvae L3).^3,4^ A key metric used for quantifying the risk of infection with mosquito-borne pathogens is the human-biting rate, which estimates the number of mosquito bites per person per day or night. When LF infections are at low levels, large numbers of mosquitoes must be dissected in order to determine infection rates.^5^ Various mosquito-sampling methods are used in entomological studies to ensure large mosquito catches, however, the methods differ in their effectiveness. Until recently, human landing catches (HLC) have been the gold standard method for effective disease transmission index assessment,^2^ but due to the ethical problem of using human subjects to bait the mosquitos, it is no longer recommended for use in most field mosquito surveillance studies.^6^ U.S. Centers of Disease Control and Prevention (CDC) light traps have been also used, baited with different attractants, or placed near occupied untreated bed nets. The trap's light bulb attracts mosquitoes from a distance and draws them in to be trapped. Other methods, such as pyre-thrum spray catches, direct aspiration, and CDC gravid traps are also used in mosquito sampling.^2–5^ Each method has shortcomings and is subject to bias, which may influence results.^6^ Gravid traps are useful tools in entomological surveillance, as they target gravid mosquitoes, those that have already fed on blood, providing an opportunity for researchers to acquire the parasite from an infected individual. In contrast, pyrethrum spray is able to knock down mosquitoes within the house where the spraying is done. Mosquito species differ in their feeding, resting, and breeding behaviours as well as their ecological requirements. As such, mosquito collection for transmission dynamics assessment requires a method or methods that can take into consideration the level of prevalence in the area and the ability of that method(s) to obtain mosquitoes of different species and physiological status. The targeted method should be the one that can capture a large number of mosquitoes with high infection and infectivity rates.

The choice of the method depends on the objectives of the study, the environment, and the available means.^2^ This study compares three methods – CDC light traps and gravid traps (John W. Hock Co., Gainesville, FL, U.S.A.) and pyre-thrum spray catches (Knockdown) – to determine the most effective for vector collection in Kenya.

## METHODS

### Study Site

**FIGURE 1.**
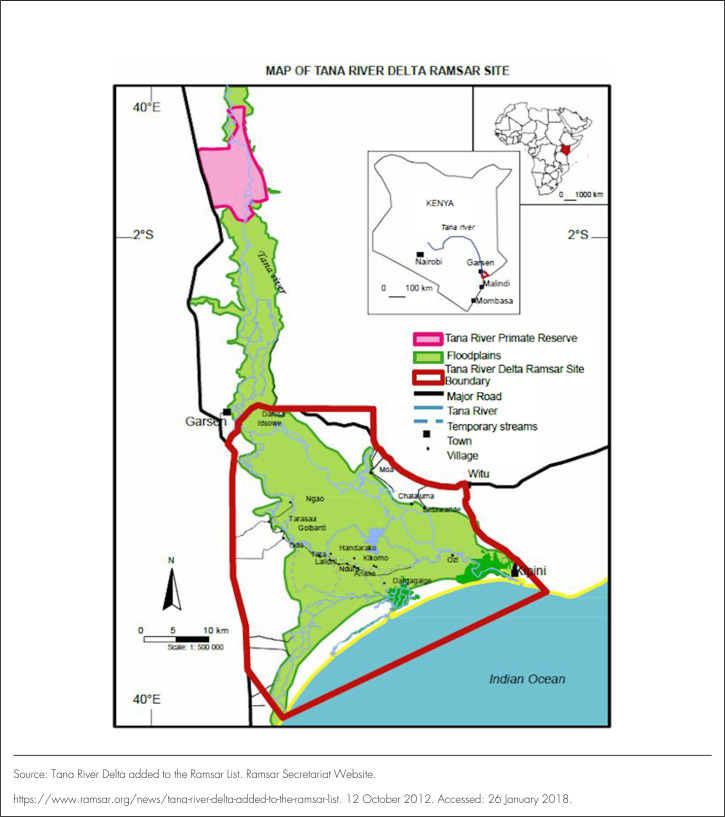
Map of Tana River/Delta, Kenya

Source: Tana River Delta added to the Ramsar List. Ramsar Secretariat Website. https://www.ramsar.org/news/tana-river-delta-added-to-the-ramsar-list. 12 October 2012. Accessed: 26 January 2018.

**Photo 1.**
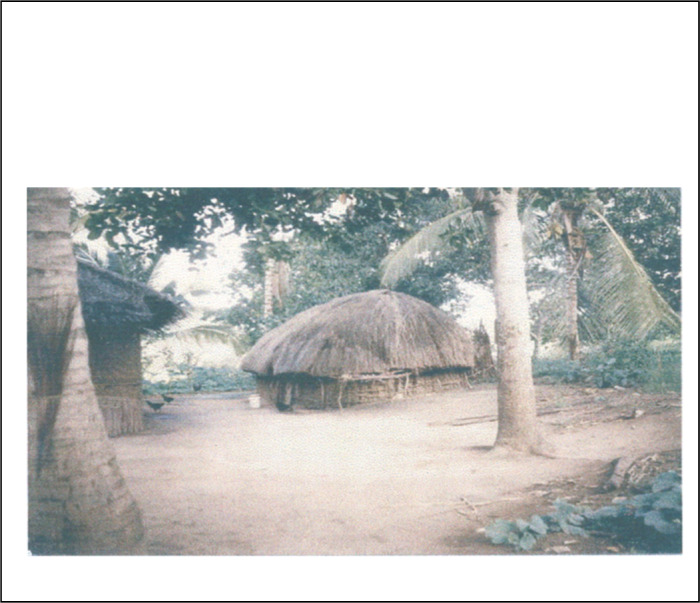
A typical house in the study area with high mosquito contact. The house is made of stick walls and grass-thatched roof.

Mosquitoes were collected from 6 villages: Kilelengwani, Hewani, Idsowe, Kisiwani farm, Onindo, and Chakamba villages of Tana River County, Coastal, Kenya (**Figure 1**). The villages were chosen based on infection prevalences and abundance of mosquitoes from previous studies.^7^ The houses were made of grass thatch both on the roof and walls, mud walled and grass-thatched houses, or block walled and galvanise iron sheet roofs with window screens. In Hewani village, most of the houses were built of burned bricks with iron sheet roofs and window screens. In Kilelengwani and Kisiwani farm, most of the houses were made of bricks with iron sheet roofs and windows without wire mesh screens. Other houses were made of bricks with a grass-thatched roof. In the rest of the villages, most of the houses were made of mud walls or sticks smeared with cow dug and grass-thatched roofs (**Photo 1**). The rainfall in this region ranges between 220 and 900 mm per year, which falls in 2 rain seasons: long rains between March and May and short rains between October and December. The major ethnic groups are the Pokomo, who practise farming and fishing, and the Orma and Wardey, who are predominantly nomadic. These farming and fishing practices create favourable mosquito breeding areas for transmission of LF.

### Study Design and Mosquito Collection

**Photo 2.**
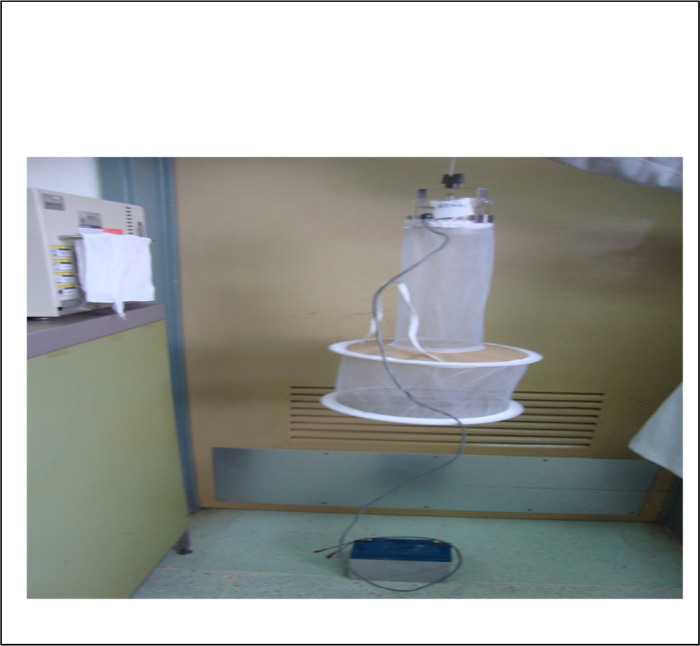
A CDC light trap set inside a house to trap mosquitoes seeking a host indoors.

**Photo 3.**
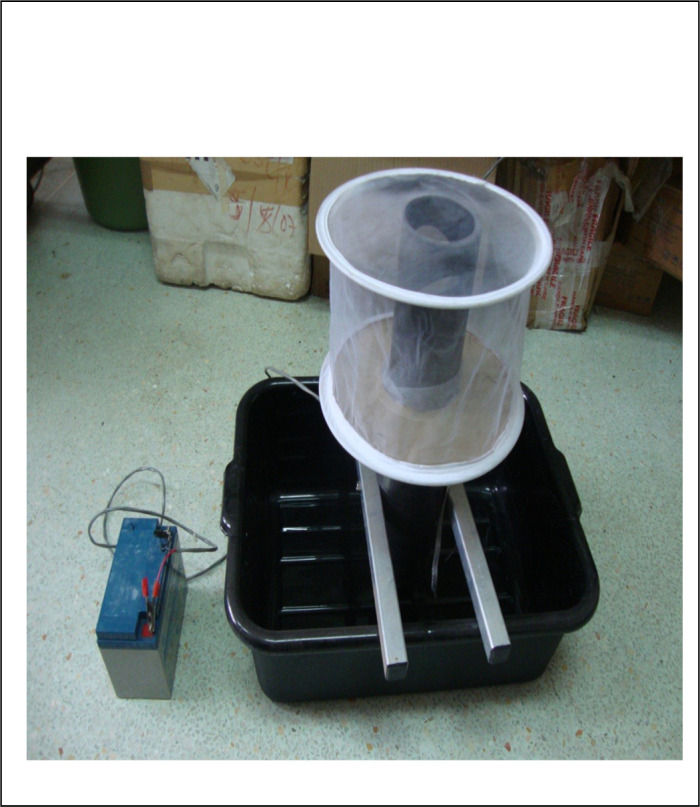
A CDC gravid trap set inside a house to trap mosquitoes seeking a host indoors.

**Photo 4.**
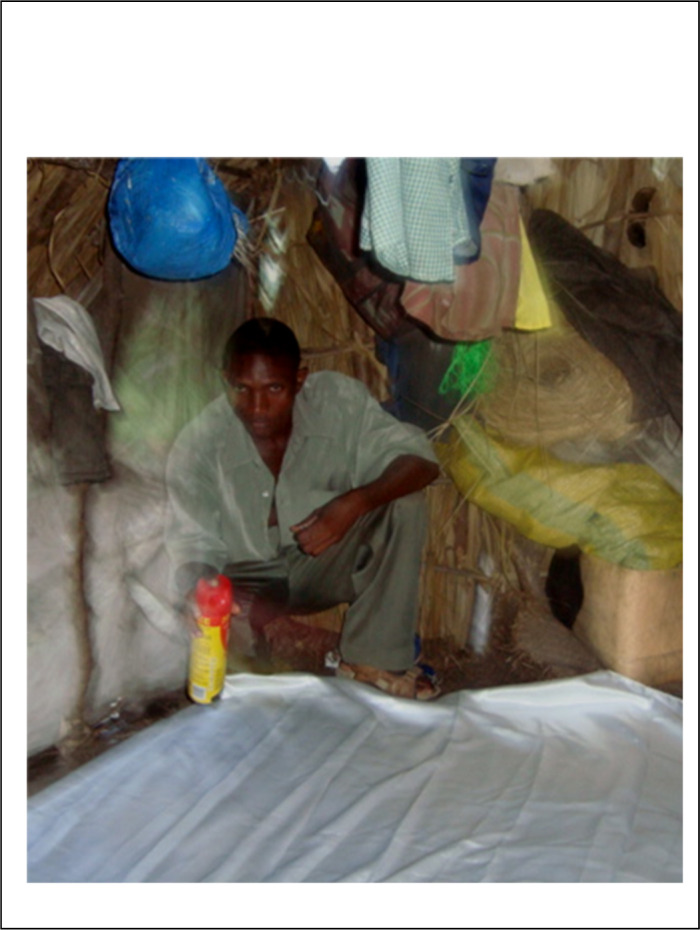
Pyrethrum spraying of the knocked down mosquitoes on a white sheet inside a house.

This was a cross-sectional study design. A total of 60 houses – 10 houses in each of the 6 study villages – were selected using simple random selection whereby the villages were assigned a number and 6 numbers were selected for mosquito sampling. Oral consent to collect mosquitoes was obtained from the area chief, household heads, and the occupants of the selected houses. Mosquitoes were collected between April and August 2010, during the long rain season, which coincides with high transmission intensity of *W. bancrofti* due to high mosquito densities.^8^ Indoor collection of mosquitoes was done using CDC light traps, gravid traps, and pyrethrum spray catches (**Photos 2**, **3**, **4**). Light traps and gravid traps were set from 7:00 pm to 7:00 am in the same houses for better comparison. Pyrethrum spray catches were done 5 days after use of the traps in the same houses. Alternation was done whereby spray catches would be done 5 days before the traps to avoid bias. Spraying was done in the evening between 7:00 pm and 10:00 pm and in the early morning between 5:00 am and 7:00 am.

### Mosquito Processing

To analyse the mosquitoes, researchers first used chloroform to kill them and then removed them from the trapping nets. All mosquitoes were counted and recorded as per the method used. The village of collection and household location were recorded on Eppendorf tubes. The mosquitoes were morphologically identified up to genera level using entomological keys.^9^ The abdominal statuses of the mosquitoes were determined as unfed, blood fed, half gravid, or gravid. All the mosquitoes were dissected to determine if microfilaria were present.

### Deoxyribonucleic Acid Amplification By Polymerase Chain Reaction Assays

Deoxyribonucleic acid (DNA) extraction was done as described by Ramzy et al^10^ with few modifications. The extracted DNA was amplified in a polymerase chain reaction (PCR) thermo cycler (GeneAmp PCR system 9700), detection and analysis of the PCR products were done through gel electrophoresis. PCR was used to amplify Ssp1 repeat of the DNA, the primer sequences were NV1 forward 5’ CAACCAGAATACCATTCATCC 3’ and NV2 reverse 5’ CGTGATGGCATAAAGTAGCG 3’ to amplify a 188-bp product in gDNA of *W. bancrofti* and the product confirmed through agarose gel electrophoresis.^10,11^ Nested PCR was carried out on a few randomly selected PCR products for results confirmation. A positive and a negative control were included in each reaction, together with a molecular weight marker for size determination.

### Data Analysis

Data were entered in record books and then transferred to a Microsoft excel spreadsheet. The data were analysed using SPSS Version 15.0 (SPSS Inc., Chicago, IL, USA). Generalized linear model univariate analysis was used to test statistical differences of the 3 methods used in collection and any other factors affecting the density of the collected mosquitoes.

### Ethical Approval

Ethical approval for this study was obtained from Kenya Medical Research Institute Scientific Steering Committee (SSC) and the National Ethical Review board (SSC protocol No. 1692).

## RESULTS

### Mosquito Collection Methods and Abdominal Status Analysis

A total of 1632 female mosquitoes were collected: 1265 (77.55%) by light traps, 311 (19.1%) by pyrethrum sprays, and 56 (3.4%) by gravid traps (**Table 1**). The mean difference of number of mosquitoes collected by the 3 methods was significant, (*P*<.0001) at 95% confidence interval (CI). While light traps were significantly different to pyre-thrum spray and the gravid methods, there was no significant difference between pyrethrum spray and gravid traps by post hoc test (*P*>.01).

**TABLE 1.** Number and Abdominal Status of the Mosquitoes Collected by Each Method

	Blood Fed				
Collection Method	Fed n (%)	Unfed n (%)	Gravid n (%)	Half Gravid n (%)	Total n
Light traps	75 (91.5)	1163 (79.6)	13 (19.1)	14 (63.6)	1265
Gravid traps	1 (1.2)	0 (0.0)	54 (79.4)	1 (4.6)	56
Pyrethroid spray	6 (7.3)	297 (20.4)	1 (1.5)	7 (31.8)	311
Total	82	1460	68	22	1632

Out of the total number of mosquitoes collected, 1460 (89.5%) were unfed, 82 (5%) were blood fed, 68 (4.2%) gravid, and 22 (1.3%) half gravid (**Table 1**). Blood-fed mosquitoes were obtained by light traps (n=75, 91.5%), pyrethrum spray (n=6, 7.3%), and gravid traps (n=1, 1.2%) (**Table 1**). Most of the gravid mosquitoes were obtained by gravid traps (n=54, 79.4%), followed by light traps (n=13, 19.1%) and pyrethrum spray (n=1, 1.5%) (**Table 1**). Almost two-thirds of half-gravid mosquitoes were obtained by light traps (n=14, 63.6%), followed by pyrethrum spray (n=7, 31.8%) and gravid traps (n=1, 4.6%) (**Table 1**). The abdominal status of the mosquitoes obtained by different methods were significantly different (*P*<.05).

### Mosquito Identification

Five mosquito genera were identified from the collected mosquitoes. Almost two-thirds (n=1048, 64.2%) were *Culex*, followed by *Mansonia* (n=236, 14.5%), *Aedes* (n=188, 11.5%), *Anopheles* (n=148, 9.1%), and *Ficalbia* (n=12, 0.7%) (**Table 2**). The species collected from the *Anopheles* genera included *An. gambiae sensu lato* (n=83, 56.0%), *An. Arabiensis* (n=33, 22.3%), *An. funestus* (n=30, 20.3%), and *An. sinensis* (n=2, 1.4%) (**Table 2**). Under *Culex* genera the species identified were *Cx. quinquefasciatus* (n=993, 94.8%) and *Cx. Pipiens* (n=55, 5.2%). The mosquitoes of *Mansonia* genera belonged to the *Mn. africanus* (n=150, 63.6%) and *Mn. Unformis* (n=86, 36.4%) species, while those of *Aedes* genera belonged to *Ae. Aegypti* (n=98, 52.1%), *Ae. Polynesiensis* (n=70, 37.2%), *Ae. Scapularis* (n=17, 9.1%), and *Ae. Mucedual sudaneses* (n=3, 1.6%) (**Table 2**).

**TABLE 2.** Mosquito Species Composition Collected in the Study Area

Anopheles		Culex		Mansonia		Aedes		Ficalbia	
Species	n (%)	Species	n (%)	Species	n (%)	Species	n (%)	Species	n (%)
*An. gambiae sl*.	83 (56.0)	*Cx. quinquefasciatus*	993 (94.8)	*Mn. africanus*	150 (63.6)	*Ae. egypti*	98 (52.1)	*Fi. uniformis theobalb*	12 (100.0)
*An. arabiensis*	33 (22.3)	*Cx. pipiens*	55 (5.2)	*Mn. unformis*	86 (36.4)	*Ae. mucedual sudaneses*	3 (1.6)		
*An. funestus*	30 (20.3)					*Ae. polynesiensis*	70 (37.2)		
*An. sinensis*	2 (1.4)					*Ae. scapularis*	17 (9.1)		
Total genera	148 (9.1)		1048 (64.2)		236 (14.5)		188 (11.5)		12 (0.7)

Abbreviations: *Ae., Aedes; An., Anopheles; Cx., Culex; Mn., Mansonia; W., Wucheria*.

### Mosquito Genera Obtained By Each of the Collection Method

Each mosquito genera appeared to have a preference for a specific collection method. Out of the 1048 (64.2%) *Culex* mosquitoes obtained, the light trap caught the highest number (n=970, 92.6%) compared to the pyrethrum spray method (n=45, 4.3%) or the gravid traps (n=33, 3.1%) (**Figure 1**). Most of the *Aedes* mosquitoes were caught by the spray method (n=176, 93.6%) compared to almost two-thirds of the *Mansonia* mosquitoes were obtained by light traps (n=153, 64.8%), compared to pyrethrum spray (n=71, 30.1%) and gravid traps (n=12, 5.1%). Almost all of the *Anopheles* mosquitoes were obtained by light traps (n=130, 87.8%), compared to the gravid traps (n=11, 7.4%) and pyrethrum spray (n=7, 4.8%). The mosquito genera obtained by each method were statistically different (*P*<.0001) at 95% CI.

### Mosquito Composition and Distribution in the Study Area

The numbers of mosquitoes obtained from each village were different. Kilelengwani village had the highest catch (n=951, 58.3%), followed by Kisiwani farm (n=320, 19.6%), Chakamba (n=225, 13.8%), Onindo (n=105, 6.4%), Idsowe (n=19, 1.2%), and Hewani (n=12, 0.7%) (**Table 3**). The villages had a significant effect on the mosquito density caught (*P*<.0001) due to different ecological factors and farming activities. For example, Kilelengwani village, located near swamps and rice-growing pads, had the highest catch, representing 58.3% (n=951) of all the mosquitoes collected. In contrast, Hewani village had the lowest catch, representing 0.7% (n=12) of the caught mosquitoes, since there were no bodies of water around the homesteads in Hewani. Regarding the 5 mosquito genera collected from the study area (**Table 2**), the *Culex* species was the most prevalent, with the highest number (n=542, 51.7%) obtained from Kilelengwani village and the least (n=7, 0.6%) from Hewani (**Table 3**). There were no *Aedes, Anopheles*, or *Ficalbia* species obtained from Idsowe village. Hewani village had no *Mansonia* and *Ficalbia* species. *Ficalbia* species mosquitoes were obtained from Kisiwani farm (n=1, 18.3%) and Kilelengwani village (n=11, 91.7%). The villages had a significant effect on mosquito species (*P*<.0001), depending on environmental factors and human activities.

**TABLE 3.** Mosquito Genera Obtained in Each of the Collection Villages in the Study Area

	Mosquito Genera					
Collection Villages	*Culex* n (%)	*Aedes* n (%)	*Mansonia* n (%)	*Anopheles* n (%)	*Ficalbia* n (%)	Total n
**Chakamba**	142 (13.5)	2 (1.1)	61 (25.8)	20 (13.5)	0 (0.0)	225
Kisiwani Farm	259 (24.7)	6 (3.2)	45 (19.1)	9 (6.1)	1 (18.3)	320
Kilelengwani	542 (51.7)	177 (94.0)	120 (50.8)	101 (68.2)	11 (91.7)	951
Onindo	84 (8.0)	2 (1.1)	5 (2.1)	14 (9.5)	0 (0.0)	105
Idsowe	14 (1.3)	0 (0.0)	5 (2.1)	0 (0.0)	0 (0.0)	19
Hewani	7 (0.6)	1 (5.5)	0 (0.0)	4 (2.7)	0 (0.0)	12
Total	1048	188	236	148	12	1632

### Mosquito Infection Status

Upon mosquito identification and dissection, microfilariae larvae L1, L2, and L3 were found in mosquitoes of the *Anopheles* and *Culex* genera. In *An. gambiae sl*., 2 mosquitoes had L2 larvae and 3 had L3 larvae; there were no L1 larvae in this species. In *An. funestus*, 2 mosquitoes had L2 larvae, no L1 or L3 larvae were present. In the *Cx. quinquefasciatus* species, 4 mosquitoes had L1 larvae, 2 had L2 larvae, and 21 mosquitoes had L3 larvae. On *W. bancrofti* DNA detection by PCR and agarose gel electrophoresis, 7 (0.4%) mosquitoes within *An. Gambiae sl*, 2 (0.1%) of *An. funestus*, and 30 (1.8%) of *Cx. quinquefasciatus* tested positive for *W. bancrofti* DNA by PCR, representing 2.3% infection rate (**Table 4**). The infection and infectivity rates by microscopy was 2.1% and 1.5% respectively, calculated as follows:

$$\begin{array}{c} \text{Infection rate}=\frac{\text{Number of mosquites carrying L1}+\text{L}2+\text{L}3}{\text{Number of dissected mosquitoes}}\times 100\\ 34/1632=2.1\%\\ \text{Infection rate}=\frac{\text{Number of mosquites carrying L3}}{\text{Number of dissected mosquitoes}}\times 100\\ 24/1632=1.5\% \end{array}$$

**TABLE 4.** Prevalence of Microfilariae in Dissected Mosquitoes and Wucheria bancrofti DNA Detection by Polymerase Chain Reaction

		Microscopy				PCR	
Mosquito Genera	Species	Larvae L1 n	Larvae L2 n	Larvae L3 n	% Infection	*W. bancrofti* DNA n	% Infection
*Anopheles*	*An. gambiae*	0	2	3	0.3	7	0.4
	*An. funestus*	0	2	0	0.1	2	0.1
*Culex*	*Cx. quinque-fasciatus*	4	2	21	1.7	30	1.8
			Total microfilaria observed: 34 Microfilaria prevalence: 34/1632 = 2.1%			Total microfilaria DNA: 39 Microfilaria prevalence: 39/1632 = 2.3%	

Abbreviations: *An., Anopheles; Cx., Culex*; DNA, deoxyribonucleic acid; PCR, polymerase chain reaction; *W., Wucheria*.

## DISCUSSION

### Comparison of Mosquito Collection Methods

The results of this study have demonstrated the use of the 3 methods: light traps, gravid traps, and pyrethrum spray for sampling disease vectors. The field evaluation of light traps, gravid traps, and pyrethrum sprays in the same ecological settings enabled the efficient comparison of these sampling methods in mosquito collection. In this study, the 3 methods were found to be significantly different, with the light trap being more significant (*P*<.0001) compared to pyrethrum spray and gravid traps using the ANOVA univariate post hoc analysis. Differences between pyrethrum spray and the gravid traps were not significant (*P*>.05). Light traps were able to obtain large numbers of mosquitoes in areas where there were large or small numbers of mosquitoes, followed by pyrethrum sprays and then gravid traps. This suggested that light traps would be the most suitable method for capturing large numbers of mosquitoes.

The results of this study were in agreement with the results of a study in Tanzania by Mboera et al,^12^ who found out that light traps collect a lot of mosquitoes because light from the bulb could attract mosquitoes from a distance. The traps in this study were set in sleeping areas/rooms to use humans as bait for attracting mosquitoes. This was done in reference to observations of Mboera et al^12^ who reported that human baits are the most efficient in attracting mosquitoes as compared to other attractants used in traps. Different odours and carbon dioxide produced by humans have attractant effect to the mosquitoes.^12^ Light traps were able to obtain mosquitoes of different abdominal status: unfed, blood fed, half gravid, and gravid. This difference in abdominal status reflected the number of mosquitoes seeking a blood meal, those that have taken a blood meal and resting for blood digestion and egg development, and those seeking for oviposition sites.^12^ The fed and half-gravid mosquitoes have high chances of having microfilaria picked during blood meal. Gravid traps collected gravid mosquitoes seeking for oviposition sites. Hay infusions (oviposition medium) used in gravid traps only attracted gravid mosquitoes, as reported by Reiter,^13^ and this limited the captured mosquitoes only to female gravid mosquitoes, which were attracted for oviposition. Bad odour from the hay infusion used in gravid traps was a problem for people sleeping in the rooms where the traps were set. New attractant media was used each day since the rotten hay infusion produced an odour that acted as a repellent to the mosquitoes (personal observation) in the study during mosquito collection. Pyrethrum sprays were capable of obtaining unfed, fed, half-gravid, and gravid mosquitoes as long as they were in reach of the sprays. The number of blood-fed mosquitoes trapped was different for each collection method, with gravid traps having the least number (1). Gravid traps attract only gravid mosquitoes ready for oviposition and, thus, the blood meal had been digested for egg development. This means that mosquitoes obtained by gravid traps had a higher chance of being infected since they had taken at least 1 blood meal. There were no unfed or male mosquitoes obtained by gravid traps. This suggests that mosquitoes obtained by gravid traps have a higher chance of being infected as compared to pyrethrum sprays, hence are suitable for assessing disease dynamics in the vectors as it has been suggested by Reiter.^13^ However the large numbers of mosquitoes required when the infection rates are low are not achievable by gravid traps due to selection bias of only gravid mosquitoes. Different sampling methods have shown varying ability in collecting mosquitoes of different abdominal conditions, which can be more informative in disease epidemiology.^14^ The methods were significantly different in obtaining mosquitoes of different abdominal status (*P*<.05). However, most of the available sampling methods for mosquito vectors have limitations associated with their use because the different species are attracted differently because of their different behaviour. Thus, in areas with different mosquito species, it is difficult to recommend a single method as the appropriate tool for trapping host-seeking mosquitoes.

Different numbers of mosquitoes were obtained from each village due to differences in ecological factors of the villages sampled. For example, more mosquitoes were obtained in villages near swampy and marshy areas compared to villages not surrounded by water bodies. The villages where the sampling of mosquitoes was carried out had a significant effect on the number of mosquitoes obtained (*P*<.0001). These findings suggest that there are more attractive ecologic niches that favour breeding of filarial vectors in villages with highest mosquito catches than the villages with few catches. This means that understanding the ecological requirements of mosquitoes is important for vector control and human-vector contact control efforts. For example, *Mansonia* species are found in submerged vegetation and the larvae attach themselves to plants.^15^ Removal of such plants through mechanical, biological, or chemical control would effectively prevent breeding of *Mansonia* species. The numbers of mosquitoes obtained by the different sampling methods were also significantly different between houses in the same villages. This suggested that the nature of houses and housing materials influenced the mosquito density. More mosquitoes were obtained from grass-thatched houses, most of which had open windows and many eaves into the houses.

There was a significant difference in mosquito species obtained from the collection villages (*P*=.046). *Culex* species had the highest number representing 64.2% (n=1048) of all the obtained mosquitoes and *Ficalbia* species was the least representing 0.7% (n=12). According to this study, the most prevalent mosquito species in Tana Delta district were *Culex*. This is in agreement with reports by Mwandawiro et al^16^ who found that *Culex* species were the main LF vectors in both urban and rural areas. Increasing urbanization, inadequate disposal, sanitation facilities, and wet season lead to increased breeding sites for LF vectors.^17^ *Culex* species breed in the foulest waters, especially in wet pit latrines,^16^ which were common in the study area. *Mansonia* species breed in submerged vegetation, which were common around swampy and marshy areas; most of the *Mansonia* species were caught in the houses near the swamps. This suggests that different mosquito species prefer different types of breeding sites. Increases in mosquito breeding coincides with a high transmission rates as reported by Kasili et al,^18^ especially during and after the long and short rain seasons. Few mosquitoes are found during the dry season with very low transmission rates.^18^

**FIGURE 2.**
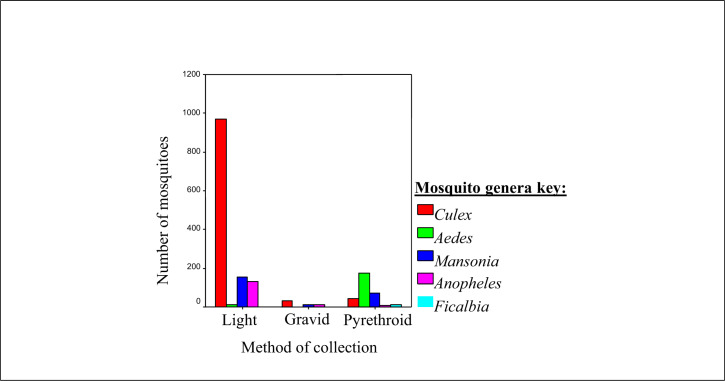
Mosquito Genera Collected by Each Sampling Method

Different genera of mosquitoes were collected by different methods. Light traps captured most of the *Culex* mosquitoes (n=970, 92.56%) (**Figure 1**), almost two-thirds of the *Mansonia* mosquito species (n=153, 64.83%), and most of the *Anopheles* mosquito species (n=130, 87.8%). The pyre-thrum spray method caught most of the *Aedes* species (n=176, 93.61%) and all the *Ficalbia* mosquitoes (**Figure 2**). No *Aedes* or *Ficalbia* mosquito species were obtained by gravid traps. The results of the mosquito species obtained by each method suggest that different methods are suitable for different mosquito species. The mosquito genera obtained by each method were significantly different (*P*<.018) at 95% CI. This information can help guide people working on mosquito vectors for different diseases, to choose the method(s) most appropriate for their specific species. For example, from this study we have determined that the spraying method would be more suitable for *Aedes* and *Ficalbia* species (**Figure 2**) and light traps are the most suitable for all species.

The results of *W. bancrofti* infection status in mosquitoes showed that mosquito species of *An. gambiae sl., An. funestus*, and *Cx. quinquefasciatus* had the capacity for transmitting *W. bancrofti* in the study area, according to dissection and *W. bancrofti* DNA detection. While the microfilaria infection rates were not significantly different using the PCR and dissection methods, however, the dissection method was labour intensive, time consuming, and tiresome as compared to PCR method.

### Study Limitations

This study did not consider comparing different seasons of the year. Data on amount of rainfall and the number of mosquitoes obtained was not gathered.

## CONCLUSIONS

In this study, light traps, were found to be the most appropriate for indoor mosquito collection, since they were capable of obtaining most mosquitoes within various genera with different abdominal status (fed, half gravid, and gravid), which have a high chance of having microfilariae. Indoor collection of mosquitoes by light traps using humans under bed nets as bait was sufficient for collecting mosquitoes needed for accurate estimation of disease transmission indices. However, combining 2 or more collection methods is ideal for accurate estimation of the disease dynamics.
